# Editorial: Eco-Sustainable Bioremediation of Textile Dye Wastewaters: Innovative Microbial Treatment Technologies and Mechanistic Insights of Textile Dye Biodegradation

**DOI:** 10.3389/fmicb.2021.707083

**Published:** 2021-07-22

**Authors:** Chirayu Desai, Kunal R. Jain, Raj Boopathy, Eric D. van Hullebusch, Datta Madamwar

**Affiliations:** ^1^Department of Biological Sciences, P. D. Patel Institute of Applied Sciences, Charotar University of Science and Technology, Changa, India; ^2^Post Graduate Department of Biosciences, Sardar Patel University, Anand, India; ^3^Department of Biological Sciences, Nicholls State University, Thibodaux, LA, United States; ^4^Université de Paris, Institut de Physique Du Globe de Paris, CNRS, UMR 7154, Paris, France

**Keywords:** textile dye, decolorization, biodegradation, bioreactors, laccase, microbial fuel cells

Textile dyeing processes utilize enormous volumes of potable water and auxiliary chemicals along with toxic dye compounds. Textile dye wastewaters are hazardous mixtures of unused textile dyes, partially degraded dye intermediates (aromatic amines) and several other xenobiotic compounds which resist biodegradation (Rathour et al., 2019). If these wastewaters are released untreated into the aquatic ecosystems, textile dyes can enter into the food webs, bioaccumulate, disrupt photosynthesis and exhibit a potential to induce ecotoxic, mutagenic and carcinogenic effects (Lellis et al., 2019). The xenobiotic and recalcitrant nature of constituent pollutants in the textile dye wastewaters makes their treatment extremely challenging. Therefore, it is imperative to devise eco-sustainable technologies for the remediation of textile dye wastewaters. Recently, various biological and physico-chemical technologies have been applied for the treatment of textile dye wastewater with varying efficacies (Deng et al., 2020). Recent research has demonstrated that microbiotechnolgy approaches, such as biodegradation of textile dyes using yeast, fungal, algal and bacterial processes are effective in the eco-friendly treatment of textile dye wastewaters (Deng et al., 2020). Similarly, enzymatic biodegradation of textile dyes by bacterial, fungal, and algal enzymes such as oxidoreductases, azo reductases, laccases, lignin peroxidases is also considered as a feasible alternative approach (Mishra and Maiti, 2019). Recent approaches utilizing bacterial communities (Rathour et al., 2019), bacterial-biofilm reactors (Rathour et al., 2021) and hybrid bioelectrochemical processes such as constructed wetland microbial fuel cells (CW-MFC) system or bacteria augmented CW-MFCs (Patel et al., 2021) have also shown promising results in the biological treatment of textile dye wastewaters. The aim of this Research Topic (RT) was to publish the recent advancements in microbial biotechnology approaches for effective bioremediation of textile dye wastewaters. This RT has published six papers including four original research papers and two mini-reviews.

The mini-review by Ceretta et al. emphasizes on an interdisciplinary approach for biological treatment of textile dye wastewater. In particular, this article provides a critical view on the state of the art of biological treatment, the degree of advancement and the prospects for their application underlining the importance of combining treatments processes while using toxicity tests on treated effluent in order to verify the toxicological quality of the treated effluents. In the mini-review by Morsy et al., various approaches used in dye decolorization processes by immobilized laccase enzymes have been summarized. The review briefly described the existing technologies based on physical, chemical and biological approaches. In the biological approach, the immobilization of laccase enzyme on solid matrix was discussed in detail with a case study on mechanism of enzyme activity of *Thermus thermophilus* HB27 (PDB code: 5JRR). Authors suggest using co-immobilization of laccase enzymes and redox mediators for the better efficiency and operational stability of the enzymes for textile dye decolorization.

In the study by Mani et al. a new approach was developed utilizing an enzymatic biocathode-MFC with *Shewanella oneidensis* MR-1 as an anodic biocatalyst for the decolorization of Acid orange 7 (AO7) dye. In this study, the *Trametes versicolor* laccase was immobilized by using three different approaches such as crosslinking with electropolymerized polyaniline (PANI), entrapment in copper alginate beads (Cu-Alg), and encapsulation in Nafion micelles, in the absence of redox mediators. Comparative analysis in this study found that biocathodes with laccase cross-linked with PANI were most suitable for efficient dye decolorization, enzyme activity retention, power production and reusability in the enzymatic-biocathode MFC systems. The paper by Dai et al. used recombinant (rlac1338) and mutant laccase (lac2-9) for decolorization of various dye compounds. The error-prone PCR approach was used to induce mutation in rlac1338 and four mutant enzymes were obtained, among which lac2-9 showed the highest activity. The observed results suggested that the expression of mutant enzyme increased by 22 ± 2% with an increase in the specific enzymatic activity. This study suggested that the error-prone PCR can be utilized in order to improve the catalytic efficiency of laccase or other dye decolorizing enzymes.

In a research paper, John et al., demonstrated the decolorization and degradation potential of halophilic bacterial strain of *Salinivibrio kushneri* HTSP for three dyes: Safranin, Congo red and CBB G-250. The bacterium under experimental conditions decolorized nearly 80% of CBB G-250 and Congo red at a wide range of dye concentrations within 48 h of incubation. They observed complete decolorization of Safranin at lower concentration (<150 mg/L), however the decolorization decreased at higher dye concentrations. Twelve different genes involved in dye degradation were annotated in the genome sequence of *Salinivibrio kushneri* HTSP. Authors conclude that, *Salinivibrio kushneri* HTSP strain has a potential to be used in textile dye wastewater treatment, however biotoxicity studies should be performed before its large-scale application. The research of Zaveri et al. utilized sodium benzoate as a model system to simulate the biodegradation of textile wastewater pollutants by *Pseudomonas citronellolis*. The study investigates how an experimental design approach enabled to understand the interplay of additional carbon and nitrogen sources as well as micronutrients on sodium benzoate degradation by *P. citronellolis*. This work clearly underlines the importance of correcting the nutrient balance in order to ensure an efficient biodegradation of aromatic pollutants present in textile effluents.

This RT highlights the bacterial biodegradation of textile dyes and integrated biological treatment of textile dye wastewater. In particular, the advancements made in enzymatic bioremediation such as biochemical characteristics of recombinant and mutant laccase enzymes, methods of immobilizing laccase enzymes and their applications in the decolorization of synthetic textile dyes as well as in bio-cathode enzymatic microbial fuel cells. This RT also addresses the current limitations, research gaps and potential solutions for future research in achieving sustainable bioremediation of textile dye wastewaters.

## Author Contributions

All the authors of this editorial article have contributed in conceptualization, writing, reviewing, revising, and have approved it for publication.

## Conflict of Interest

The authors declare that the research was conducted in the absence of any commercial or financial relationships that could be construed as a potential conflict of interest.

## References

[B1] Deng D. Lamssali M. Aryal N. Ofori-Boadu A. Jha M. K. Samuel R. E. (2020), Textiles wastewater treatment technology: a review. Water Environ. Res. 92, 1805–1810. 10.1002/wer.1437 32790931

[B2] Lellis B. Fávaro-Polonio Z. C. Pamphile J. A. Polonio J. C. (2019). Effects of textile dyes on health and the environment and bioremediation potential of living organisms. Biotechnol. Res. Innov. 3, 275–290. 10.1016/j.biori.2019.09.001

[B3] Mishra S. Maiti A. (2019). Applicability of enzymes produced from different biotic species for biodegradation of textile dyes. Clean Techn. Environ. Policy 21, 763–781. 10.1007/s10098-019-01681-5

[B4] Patel D. Bapodra S. L. Madamwar D. Desai C. (2021). Electroactive bacterial community augmentation enhances the performance of a pilot scale constructed wetland microbial fuel cell for treatment of textile dye wastewater. Bioresour. Technol. 322:125088. 10.1016/j.biortech.2021.125088 33839511

[B5] Rathour R. Jain K. Madamwar D. Desai C. (2019). Microaerophilic biodegradation of raw textile effluent by synergistic activity of bacterial community DR4. J. Environ. Manage. 250:109549. 10.1016/j.jenvman.2019.109549 31545178

[B6] Rathour R. Jain K. Madamwar D. Desai C. (2021). Performance and biofilm-associated bacterial community dynamics of an upflow fixed-film microaerophilic-aerobic bioreactor system treating raw textile effluent. J Clean. Prod. 295:126380. 10.1016/j.jclepro.2021.126380

